# Preparation and Properties of 2D Materials

**DOI:** 10.3390/nano10040764

**Published:** 2020-04-16

**Authors:** Byungjin Cho, Yonghun Kim

**Affiliations:** 1 Department of Advanced Material Engineering, Chungbuk National University, Chungdae-ro 1, Seowon-Gu, Cheongju, Chungbuk 28644, Korea; 2 Materials Center for Energy Convergence, Surface Technology Division, Korea Institute of Materials Science (KIMS), 797 Changwondaero, Sungsan-gu, Changwon, Gyeongnam 51508, Korea

Since the great success of graphene, atomically thin layered nanomaterials—called two-dimensional (2D) materials—have attracted tremendous attention due to their extraordinary physical properties. In particular, van der Waals heterostructured architectures based on a few 2D materials, named atomic scale Lego, have been proposed as unprecedented platforms for the implementation of versatile devices with a completely novel function or extremely high performance, shifting the research paradigm in materials science and engineering [1]. Thus, diverse 2D materials beyond existing bulk materials have been widely studied for promising electronic, optoelectronic, mechanical, and thermoelectric applications. In particular, this Special Issue includes the recent advances in unique preparation methods, such as exfoliation-based synthesis and the vacuum-based deposition of diverse 2D materials, as well as their device applications based on their interesting physical properties. This editorial consists of the following two sections: Preparation Methods of 2D Materials and Properties of 2D Materials.

## 1. Preparation Methods of 2D Materials

Solution-based exfoliation methods for two-dimensional (2D) materials have been intensively investigated due to the ease of the process. In this regard, Zhang et al. investigated the cost-effective exfoliation method of multilayered 2D MoS_2_ nanosheets and quantum dots from natural SiO_2_-containing molybdenite in different solutions under mild ultrasonic conditions [2]. This simple method provides several advantages such as high yields, low cost and large-scale industrial perspectives compared with conventional methods. 2D-MoS_2_ nanosheets with dimensions of 50–200 nm were prepared. Furthermore, the excellent photoconductivity of the nanosheets under visible light was demonstrated in various solution conditions. Meanwhile, the conventional method to prepare saturable absorber materials uses the Langmuir–Blodgett (LB) technique, the merits of which include its low cost. In this respect, Wang et al. demonstrated a low-cost reflective WS_2_ saturable absorber (SA) on a silver-coated mirror for the first time [3]. By using the simple LB method, a large-area and highly uniform 2D-WS_2_-coated SA was successfully shown. Moreover, the optical saturation properties of WS_2_ SA were thoroughly analyzed, with the duration being around 409 ns and the highest peak power being 5.2 W. Thus, highly reflective WS_2_ SA, created using the simple LB method, could be used in a diverse optical modulator with a wavelength of 1.3 μm.

MoO_3_ is a promising material with well-recognized applications such as electronics, photocatalysis, electrocatalysis, batteries, and pseudocapacitors [4]. Among the various crystal structures of MoO_3_, the orthorhombic α-MoO_3_ provides unique 2D morphologies with layered structures. α-MoO_3_ has been conventionally obtained via the hydrothermal method or sputtering. However, such conventional preparation methods have faced some critical challenges related to substantial energy, complex equipment, and expert operational skills. Thus, Ramos et al. report a new preparation method to obtain highly crystalline α-MoO_3_ using vapor-phase synthesis [5]. They obtained highly ordered multilayer α-MoO_3_ from molybdate using carbon nitride (g-C_3_N_4_) with a lamellar template. This simple method may be applied to electrocatalytic hydrogen evolution and ultrasensitive plasmonic biosensing.

For two-dimensional transition metal dichalcogenides (TMDCs), a uniform growth technique is required, especially for applications in electronics and optoelectronics. However, several critical challenges such as high growth temperature, limited growth area, and layer controllability still remain. Thus, Zhong et al. reported the simple growth method of 2D-MoS_2_ using a two-step process, combining radio frequency (RF) magnetron sputtering and the subsequent sulfurization process [6]. The growth temperature of this two-step process is lower than 600 °C, and the crystalline qualities are simply controlled by RF sputtering power. As RF plasma power increases from 10 to 150 W, the crystalline quality also increases, which is confirmed by the intrinsic peak intensities of the Raman spectrum. Recently, a new family of 1D nanomaterials with weak van der Waals interactions was also reported. Kim et al. successfully demonstrated the synthesis of a 1D semiconductor V_2_Se_9_ crystal using mass production via the simple transport preparation method [7]. The 1D-V_2_Se_9_ crystal exhibited weak van der Waals interaction and a nanoribbon structure. Also, scanning Kelvin probe microscopy (SKPM) analysis showed a variation in work function depending on the thickness of the V_2_Se_9_ crystal. This mass-production preparation method for 1D nanomaterials such as V_2_Se_9_ could be suitably applied to the metal contact of future van der Waals-based nanoelectronic devices.

## 2. Properties of 2D Materials

The electrical properties of 2D semiconducting materials are usually validated via the demonstration of field effect transistor (FET) devices. Thus, research themes involving the performance enhancement of FET devices have long attracted great attention, especially with respect to device junction optimization. In this context, Lim et al. proposed a novel FET structure consisting of a 2D MoS_2_/black phosphorous (BP) heterojunction, which shows a high on/off ratio of over 1 × 10^7^, along with an extremely low subthreshold swing value of ~54 mV/dec and very low off current of ~fA level [8]. Interestingly, the low off current was attributed to the depletion region in the BP layer. Meanwhile, a TiO_2_ interfacial layer inserted between a metal and 2D TMDCs (MoS_2_ and WS_2_) can also lead to enhanced FET properties [9]. In addition, a stable electrical performance could be achieved under a gate bias stress condition, since the TiO_2_ interfacial layer serves as a Fermi level depinning layer, which reduces the density of the interface states.

The synthesis of p-type MoS_2_ is often essential for the complementary integration process using p- and n-type 2D materials. Lee et al. reported that p-type semiconducting characteristics can be obtained via the addition of a dopant precursor of phosphorous pentoxide during the chemical vapor deposition synthesis process of MoS_2_ [10]. The p-doped monolayer MoS_2_ showed p-type conduction with a relatively low field effect mobility of 0.023 cm^2^/V⋅s and an on/off current ratio of 10^3^, compared with the pristine n-type MoS_2_. The performance of the p-doped FET should be further improved. Along with neuron devices, artificial synapse devices have been recently considered as one of the most essential components in implementing a neuromorphic hardware system. Finding a physical parameter that precisely modulates synaptic plasticity is particularly required. Following this motivation, Kim et al. proposed a novel two-dimensional transistor architecture consisting of a NbSe_2_/WSe_2_/Nb_2_O_5_ heterostructure [11]. NbSe_2_, WSe_2_, and Nb_2_O_5_ function as a metal electrode, an active channel, and a conductance-modulating layer, respectively. Notably, the post-synaptic current was successfully modulated by the thickness of the interlayer Nb_2_O_5_, whose introduction facilitated the realization of reliable and controllable synaptic devices.

The unique optical properties of the 2D materials were intensively investigated. For instance, using thin semiconductor MoS_2_/ferroelectric lead zirconate titanate heterostructure films, reversibly tunable photoluminescence was demonstrated during ferroelectric polarization reversal using nanoscale conductive atomic force microscopy tips [12]. The spontaneous polarization of the ferroelectric thin films affects the optoelectronic behaviors of MoS_2_ indirectly via reversible electrochemical processes. Meanwhile, the Raman spectrum of BP transferred onto a germanium-coated polydimethylsiloxane flexible substrate was systematically studied [13]. The Raman spectra obtained from several BP layers with different thicknesses showed the clear peak shifting rates for the Ag^1^, B^2^g, and Ag^2^ modes. A study of the strain–Raman spectrum relationship was also conducted, showing a maximum uniaxial strain of 0.89%. The peak shifting of Ag^1^, B_2_g, and Ag^2^ caused by this uniaxial strain was clearly measured. In another optical study, a systematic investigation of photoluminescence (PL) and Raman spectroscopy of the transferred bilayer-stacked MoS_2_ were conducted, and compared with freestanding monolayer MoS_2_ [14]. The interlayer difference and spatial inhomogeneity of exciton and phonon performance are attribute to film–substrate coupling-induced strain and doping. Even surface fluctuations with a thickness of less than one atom layer could be easily identified by Raman and PL spectroscopy, offering useful information about the 2D van de Waals homostructure and heterostructures’ effects on the optical properties of 2D materials.

The mechanical properties of the 2D materials are also interesting and attractive. In this regard, the tribological performance of two kinds of WS_2_ nanomaterials as additives in paraffin oil was investigated, showing that the friction and wear performance of paraffin oil can be greatly improved with the addition of WS_2_ nanomaterials, and that the morphology and content of WS_2_ nanomaterials have a significant effect on the tribological properties of paraffin oil [15]. For instance, paraffin oil with WS_2_ nanoflowers exhibited better tribological properties than that with WS_2_ nanoplates. The superior tribological properties of the WS_2_ nanoflowers were attributed to their special morphology, which contributes to the formation of a uniform tribofilm during the sliding process.

We hope this Special Issue will help 2D material researchers follow up the latest research trends and progress in the 2D research community.
